# Fibrillin-1 Mutations Causing Weill-Marchesani Syndrome and Acromicric and Geleophysic Dysplasias Disrupt Heparan Sulfate Interactions

**DOI:** 10.1371/journal.pone.0048634

**Published:** 2012-11-02

**Authors:** Stuart A. Cain, Amanda McGovern, Andrew K. Baldwin, Clair Baldock, Cay M. Kielty

**Affiliations:** Wellcome Trust Centre for Cell-Matrix Research, Faculty of Life Sciences, University of Manchester, Manchester, United Kingdom; Indiana University School of Medicine, United States of America

## Abstract

The extracellular glycoprotein fibrillin-1 forms microfibrils that act as the template for elastic fibers. Most mutations in fibrillin-1 cause Marfan syndrome with severe cardiovascular and ocular symptoms, and tall stature. This is in contrast to mutations within a heparin-binding TB domain (TB5), which is downstream of the arg-gly-asp cell adhesion domain, which can cause Weill-Marchesani syndrome (WMS) or Acromicric (AD) and Geleophysic Dysplasias (GD). WMS is characterized by short limbs, joint stiffness and ocular defects, whilst fibrillin-1 AD and GD have severe short stature, joint defects and thickened skin. We previously showed that TB5 binds heparin. Here, we show that the corresponding region of fibrillin-2 binds heparin very poorly, highlighting a novel functional difference between the two isoforms. This finding enabled us to map heparin/heparan sulfate binding to two sites on fibrillin-1 TB5 using a mutagenesis approach. Once these sites were mapped, we were able to investigate whether disease-causing mutations in this domain disrupt binding to HS. We show that a WMS deletion mutant, and five AD and GD point mutants all have disrupted heparin binding to TB5. These data provide insights into the biology of fibrillins and the pathologies of WMS, AD and GD.

## Introduction

Fibrillin-1 (FBN1) is a major glycoprotein of the extracellular matrix, which contains repeating calcium-binding epidermal growth factor (cbEGF)-like domains interspersed with eight-cysteine (TB) domains [1]. It assembles pericellularly to form microfibrils that support the deposition of elastin in tissues such as blood vessels and skin [2], [3]. Fibrillin-1 binds heparin/heparan sulfate (HS) at multiple sites, with high affinity binding to multimeric fibrillin-1 [4]–[7]. We and others have shown that fibrillin-1 also supports cell adhesion through its arg-gly-asp (RGD) motif within the fourth TB domain (TB4) [8]–[12]. We also showed that adjacent TB5 binds heparin [9].

Numerous mutations in fibrillin-1 cause Marfan syndrome, a severe inherited disease associated with long limbs and major cardiovascular and lens defects [13]. In contrast, two fibrillin-1 mutations cause autosomal dominant Weill-Marchesani syndrome (WMS), which is characterized by short stature, thick skin, stiff joints and ocular problems [14], [15]. One of these mutations is an in-frame deletion within TB5 [14]. In addition, 16 point mutations have been reported in autosomal dominant Acromicric Dysplasia (AD) and Geleophysic Dysplasia (GD), which also cause severe short stature, join defects and thickened skin [16]. It has remained unclear why mutations in TB5 cause WMS, AD or GD rather than Marfan syndrome.

Here, we have mapped two heparin-binding sites within the fibrillin-1 TB5 domain and shown that the corresponding domain of fibrillin-2 does not bind heparin. We have also demonstrated that WMS, AD and GD disease-causing mutations have disrupted heparin binding by TB5. The study thus sheds light on the biology of fibrillins and the pathogenesis of WMS, AD and GD.

## Materials and Methods

### Recombinant Fibrillin-1 and Fibrillin-2 Fragments

**Figure 1:**
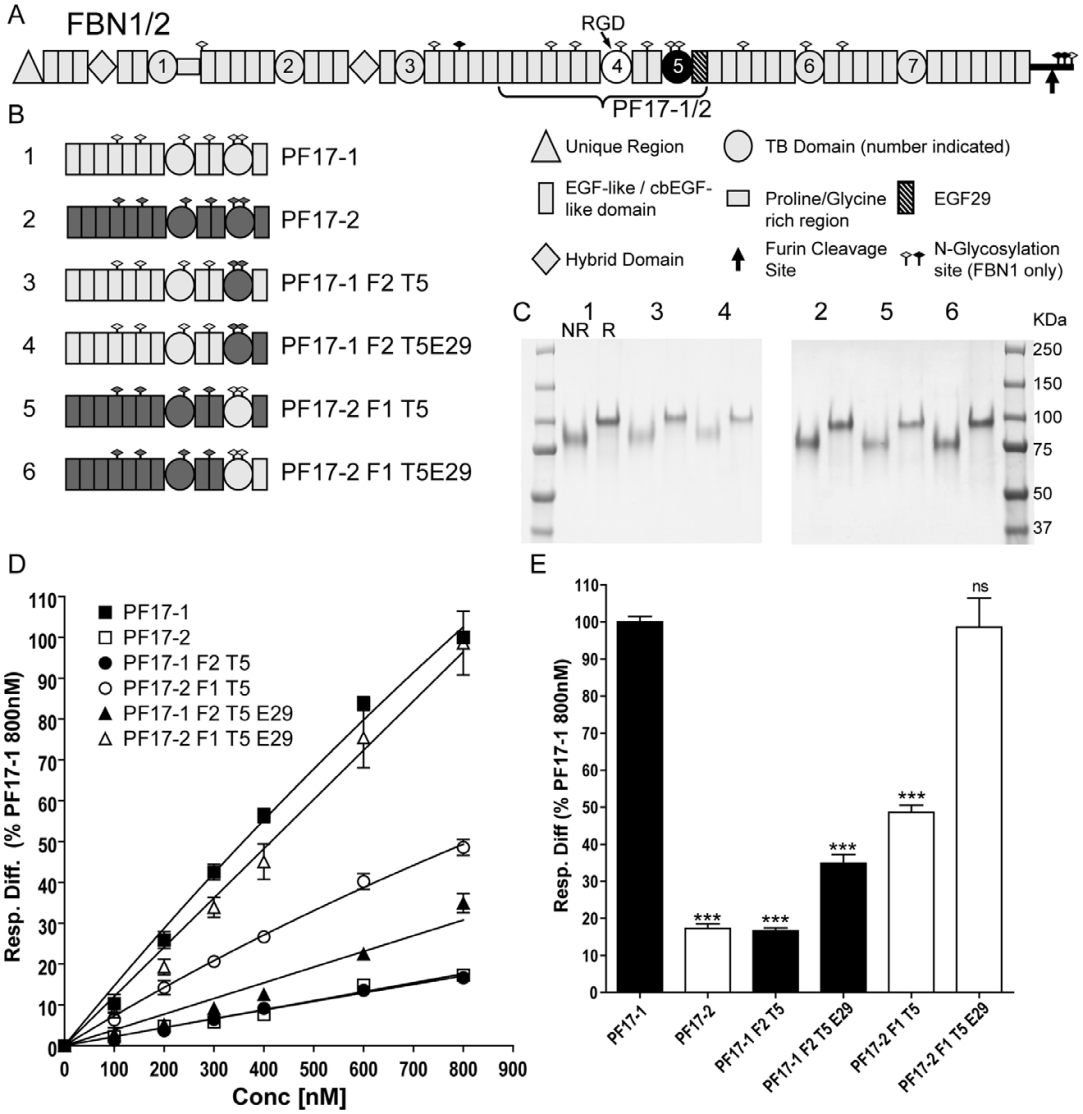
Schematic of recombinant fibrillin-1 and fibrillin-2 domain swap fragments and their heparin binding properties. (A) Domain structures of fibrillin-1 and fibrillin-2 are shown, with a key of the different domains, N-glycosylation sites, and the C-terminal furin cleavage site. Fibrillin-1 contains a proline-rich region and fibrillin-2 contains a glycine-rich region. A protein fragment containing the cell adhesion RGD site (indicated) of TB4 (white fill) and heparin binding site of TB5 (black fill) was produced for fibrillin-1 (PF17-1) and fibrillin-2 (PF17-2). (B) All protein fragments used in this study are shown indicating which domains have been swapped. Fibrillin-1 domains are shown in light grey and Fibrillin-2 domains shown in dark grey, along with the number indicating the position on SDS-PAGE. (C) SDS-PAGE of all PF17-1 and PF17-2 fragments run under non-reducing (NR) and reducing conditions (R). All proteins run between the 75 and 100 Kda protein markers. (D) Heparin binding of PF17-1, PF17-2 and PF17-1/2 domain swaps. Binding was analyzed using Surface Plasmon Resonance, and the response difference (Resp. Diff.) normalized to PF17-1 response level at 800 nM for each experiment and was plotted against concentration. Resp. Diff. is the heparin-immobilized flow cell minus the control flow cell. The value shown is average normalized response difference and SEM of three separate experiments. Representative sensorgrams are shown in Figure S1. (**E**) Graph plotting the average normalized response difference to PF17-1 and SEM at 800 nM of at least three separate experiments. Also shown are the statistical significance of the difference to PF17-1 where P value = >0.05 ns; <0.001 ***.

Recombinant human wild-type and mutant fibrillin-1 and fibrillin-2 fragments were expressed using the mammalian expression vector pCEP-pu/AC7 modified with a six histidine tag and 293-EBNA cells [6]–[9]. All constructs expressed well and all recombinant proteins were efficiently secreted. Fibrillin-1 PF17-1 is encoded by exons 30–43 (residues 1238–1807). Fibrillin-2 was cloned from RNA extracted from human dermal fibroblasts (HDFs); PF17-2 is encoded by exons 30–43 (residues 1283–1849). Four domain swap constructs were produced using overlap PCR, where in the first round of PCR either 2 or 3 separate fragments were generated depending on the construct, each containing overlapping sequences. These fragments were combined in a second round of PCR to create each construct. A schematic of the process is shown (Fig. S1A). The four domain swap constructs were: PF17-1 F2 T5, which was the fibrillin-1 PF17-1 construct containing fibrillin-2 TB5; PF17-1 F2 T5E29 which was the fibrillin-1 PF17-1 construct containing fibrillin-2 TB5 and the following EGF29; PF17-2 F1 T5 which was the fibrillin-2 PF17-2 construct containing fibrillin-1 TB5; and PF17-2 F1 T5E29 which was the fibrillin-2 PF17-2 construct containing fibrillin-1 TB5 and the following EGF29 (Fig. 1A,B). All fragments were fully sequenced prior to transfection. Purity of recombinant proteins was analyzed using SDS-PAGE (Fig. 1C).

To map the fibrillin-1 PF17-1 heparin binding site, point mutations were incorporated into the fibrillin-2 PF17-2 fragment to ‘rescue’ heparin binding, using QuikChange Site-Directed Mutagenesis Kit (Agilent). PF17-2 K1737R contains the mutation at amino acid 1737 Lys to Arg; PF17-2 K1737R/F1739L contains the mutations amino acid 1737 Lys to Arg and amino acid 1739 Phe to Leu; PF17-2 K1770R contains the mutation amino acid 1770 Lys to Arg; PF17-2+QI1794R contains the mutation amino acid 1794 Ile to Arg, with an insertion of a Gln residue between amino acid 1793 and 1794. PF17-2 Quad contained all four mutations described at amino acids 1737, 1739, 1770 and 1794 and also the insertion of the Gln residue. All recombinant proteins were analyzed using SDS-PAGE (*see* Fig. S2C).

**Figure 2:**
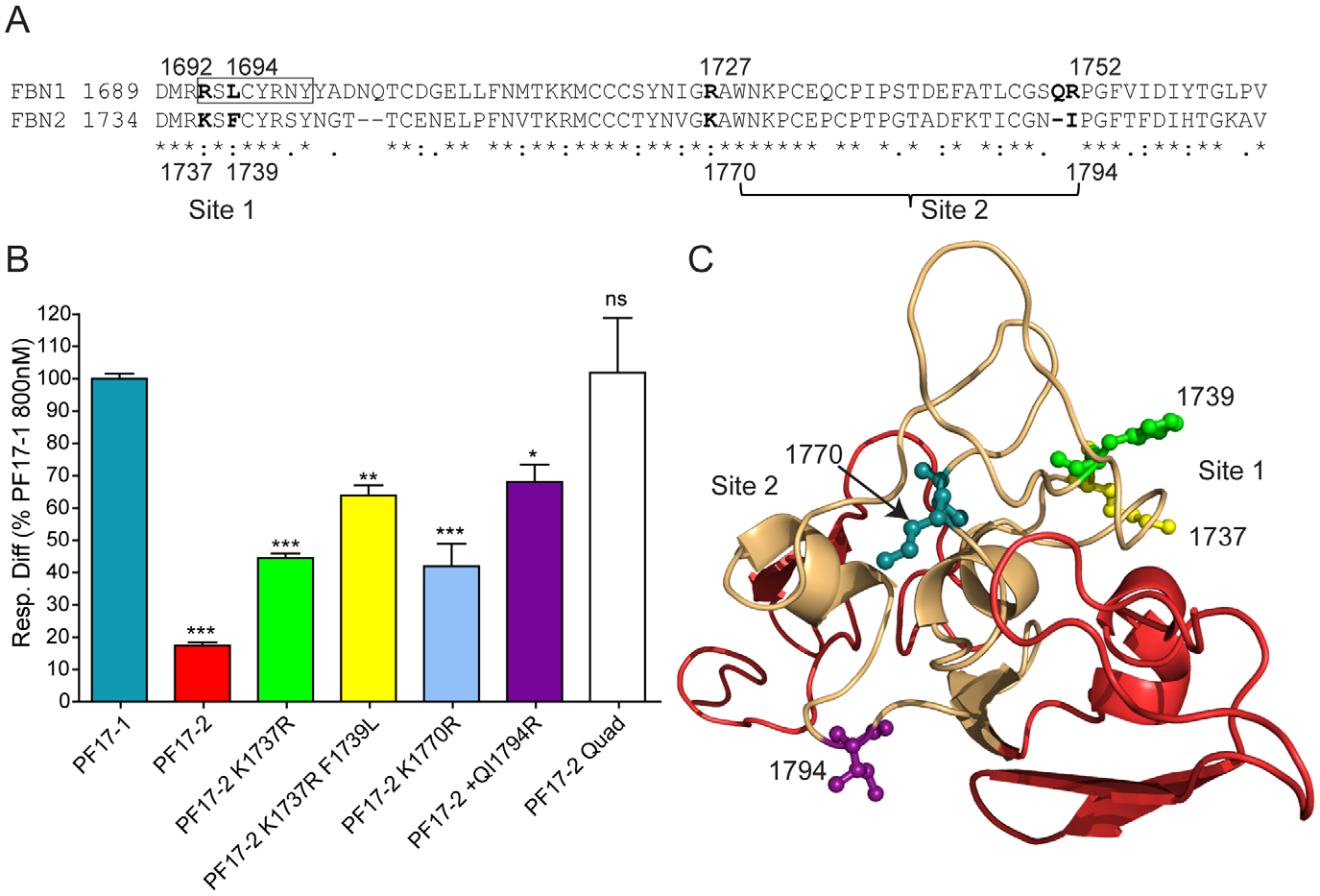
Heparin binding of the point mutations of fibrillin-2 protein fragment PF17-2. (A) Sequence alignment of human fibrillin-1 and fibrillin-2 TB5, indicating the fibrillin-1 residue positions of the two heparin binding sites (indicated), and the corresponding residue numbers of fibrillin-2. Boxed section represents the 8 amino acid WMS deletion. (B) Heparin binding of PF17-1, PF17-2 and PF17-2 point mutations, including a fragment with all four point mutations designated PF17-2 Quad. Binding was analyzed using Surface Plasmon Resonance, and the average response difference (Resp. Diff.) for three separate experiments normalized to PF17-1 response level at 800 nM for each experiment and SEM is shown. Resp. Diff is the heparin-immobilized flow cell minus the control flow cell. Also shown are the statistical significances of the difference to PF17-1 where P value >0.05, ns; <0.05, *; <0.01, **; <0.001, ***. A representative sensorgram for all concentrations and affinity plots is shown in Figure S2. (C) Structural model of fibrillin-2 TB5 (beige) and anterior/posterior EGF domains (red), showing the position of the point mutations, which fall on either side of the TB domain, indicating the two heparin binding sites. A structural model was created using SwissModel, as described in Material and Methods.

Disease-causing fibrillin-1 mutations were also inserted into PF17-1. PF17-1 WMS contained the 8 amino acid deletion found in patients with WMS [14] (amino acids 1692–1699 RSLCYRNY), and was constructed using overlap PCR. This fragment was fully sequenced prior to transfection, and the presence of the deletion mutation was confirmed. Six further fibrillin-1 mutations found in patients with GD and AD [16] were inserted into PF17-1. The AD and GD point mutants which did not originally contain cysteine residues that were chosen were Y1696C [GD], Y1699C [GD/AD], M1714R [AD], G1726V [AD], A1728T [GD/AD], S1750R [AD]; all were generated by site-directed mutagenesis. All PCR primers used are shown in Table S1.

**Figure 3:**
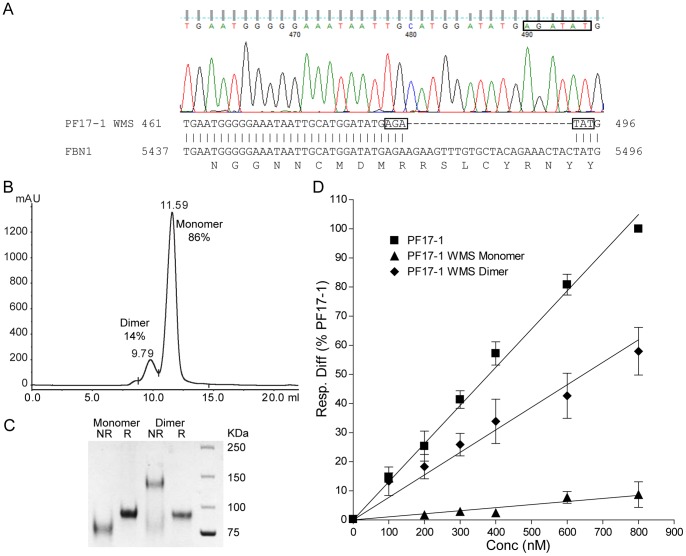
Heparin binding of the WMS mutant of fibrillin-1 protein fragment PF17-1. ( A) Sequence trace from mammalian expression vector pCEP-pu/AC7 containing fibrillin-1 PF17-1 WMS mutant. The sequence trace generated by Finch TV V1.4 with the two codons either side of the 24 bp deletion (boxed) is shown. Below is a section of the resulting Blast search showing the alignment with human fibrillin-1, indicating the same two codons. Also shown is the corresponding amino acid sequence aligned with the last base pair of each codon. (B) Chromatographic trace of size exclusion chromatography of PF17-1 WMS fragment using Superdex S200 10/300 GL column (GE Healthcare), showing peaks and retention volume of monomer and dimer of PF17-1 WMS. (C) SDS-PAGE of all PF17-1 WMS monomer and dimer fragments run under non-reducing (NR) and reducing conditions (Red). (D) Heparin binding of PF17-1, PF17-1 WMS monomer and dimer protein fragments. Binding was analyzed using Surface Plasmon Resonance, and the response difference (Resp. Diff.) normalized to PF17-1 response level at 800 nM for each experiment was plotted against concentration. Resp. Diff. is the heparin-immobilized flow cell minus the control flow cell. The value shown is average normalized Resp. Diff. and SEM of three separate experiments. Representative sensorgrams are shown in Figure S3C.

For cell spreading assays, two shorter fibrillin-1 fragments were also used. Fibrillin-1 fragments FBN1 PF8 (encoded by exons 30–38 (residues 1238–1605)), which contains the RGD motif but not heparin binding site, and FBN1 PF10 (encoded by exons 41–52 (residues 1688–2165)), which contains the heparin binding sites but not the RGD motif, were expressed using the same methods and have been described previously [17].

**Figure 4:**
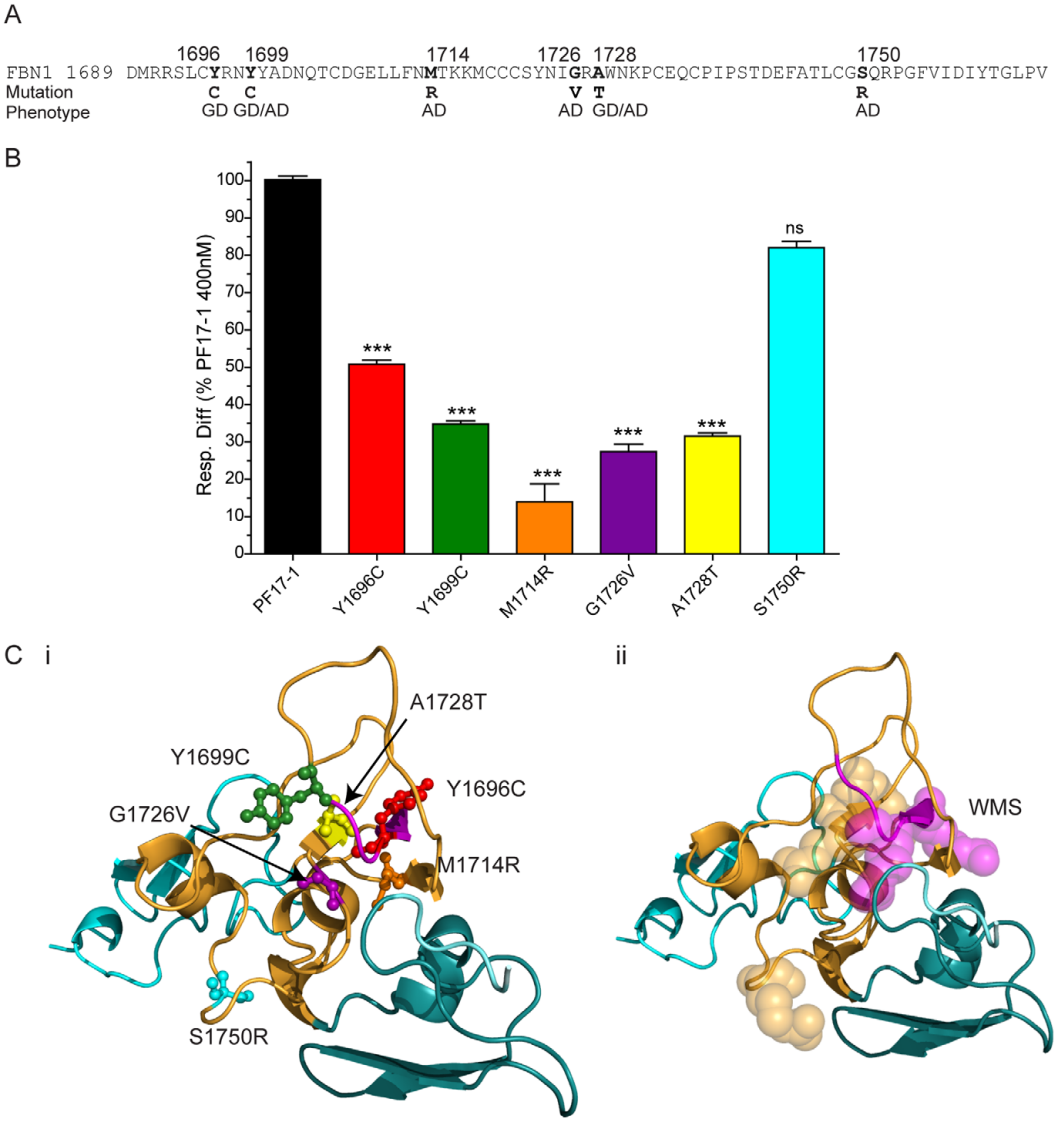
Heparin binding of the GD/AD mutants of fibrillin-1 protein fragment PF17-1. (A) Sequence of human FBN 1 TB5, indicating the fibrillin-1 residue positions of the GD/AD point mutations along with their corresponding phenotype. (B) Heparin binding of PF17-1, and the GD/AD point mutations. Binding was analyzed using surface plasmon resonance, and the average response difference (Resp. Diff.) for three separate experiments normalized to PF17-1 response level at 400 nM for each experiment and SEM is shown. Resp. Diff is the heparin-immobilized flow cell minus the control flow cell. Also shown are the statistical significances of the difference to PF17-1 where P value >0.05, ns; <0.001, ***. (C) Structural model of fibrillin-1 TB5 (orange) and anterior/posterior cbEGF domains (cyan), showing the position of the GD/AD point mutations (i). Also shown (ii) is the same structural model indicating the region of the WMS deletion (magenta) and the Arg residues implicated in heparin binding (shown as spheres). The structural model was created using SwissModel as described in Material and Methods.

In all cases, monomeric fragments were purified using nickel affinity chromatography and monomeric species for each fragment were further purified by gel filtration chromatography using a Superdex 200 10/300 GL column (GE Healthcare), as described [7]. Using validated monomers enabled comparisons between fragments. Molecular masses of purified monomeric fragments were analyzed using Multi Angle Laser Light Scattering (MALLS) using a Wyatt EOS 18-angle light scattering detector fitted with a 688-nm laser and an Optilab r-EX refractometer as described [7].

**Figure 5:**
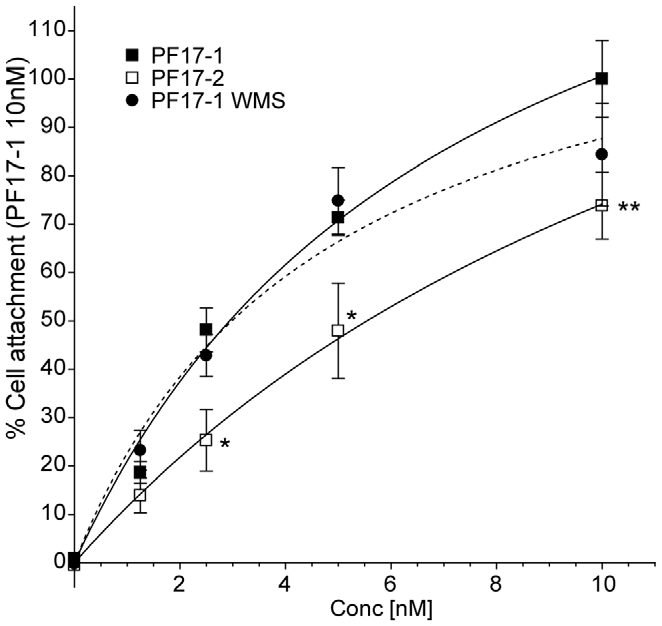
Cell attachment of human dermal fibroblasts to fibrillin-1 and fibrillin-2 protein fragments. (A) Cell attachment of human dermal fibroblasts (HDF) to PF17-1, PF17-2 and PF17-1 WMS. HDF were added to cell culture plate wells pre-incubated with increasing concentrations of protein fragments (0–10 nM), for 1 hour. After removal of non-adhered cells, adhered cells were stained with crystal violet and the optical densities (OD) at 570 nm were measured as described in Materials and Methods. Values were normalized to the percentage of cell attachment of PF17-1 at 10 nM. Also shown are the statistical significances of the difference to PF17-1 where P value <0.05, *; <0.01, **.

### Biacore Analysis of PF17-1 and PF17-2 Fragment Interactions with Heparin Oligosaccharide

For the kinetic binding studies, a heparin oligosaccharide consisting of 20 sugar moieties (dp20) (Iduron, UK) was biotinylated via oxidized cis-diol groups and immobilized onto SA sensor chips (GE Healthcare), as described [6]. The heparin oligosaccharide was used at 1 µM, and 500 Response Units (RU) were immobilized. All binding experiments were performed in 10 mM HEPES pH 7.4, 0.1 M NaCl, 0.5 mM CaCl_2_ and 0.005% surfactant P20 (designated HBS-P). Although multimeric fibrillin-1 binds heparin much more strongly than monomers [4]–[7], we used only verified monomeric PF17-1 and PF17-2 fragments in order to exclude variable levels of aggregates and to ensure reliable comparison between wild-type and mutant fragments. Fragments were injected, at concentrations ranging from 0–800 nM, at a flow rate of 30 µl/min for 6 minutes, and dissociated for 10 minutes. Regeneration was performed by two 30s injections of 0.5 mM NaOH 1 M NaCl. Curves were initially fitted using the 1∶1 Langmuir association/dissociation model (BIAevaluation 4.1, GE Healthcare), however model fitting of the curves and K_D_ calculations proved inaccurate, probably because the interactions involved >1 binding site on TB5 (see results). Therefore to compare different protein fragments, response levels were normalized to PF17-1 binding at 800 nM for each experimental run. To discover whether interactions were specific to heparin, biotinylated chondroitin 6-sulfate and hyaluronan were also immobilized onto SA sensor chips, as described [6]. Known hyaluronan binding proteins; G1 domain of versican and TNF-stimulated gene 6 protein (provided by Prof. A. J. Day, University of Manchester) were used as positive controls, at concentrations of 200 nM. Chondroitin sulfate immobilization using the same methodology has been reported [6].

**Figure 6:**
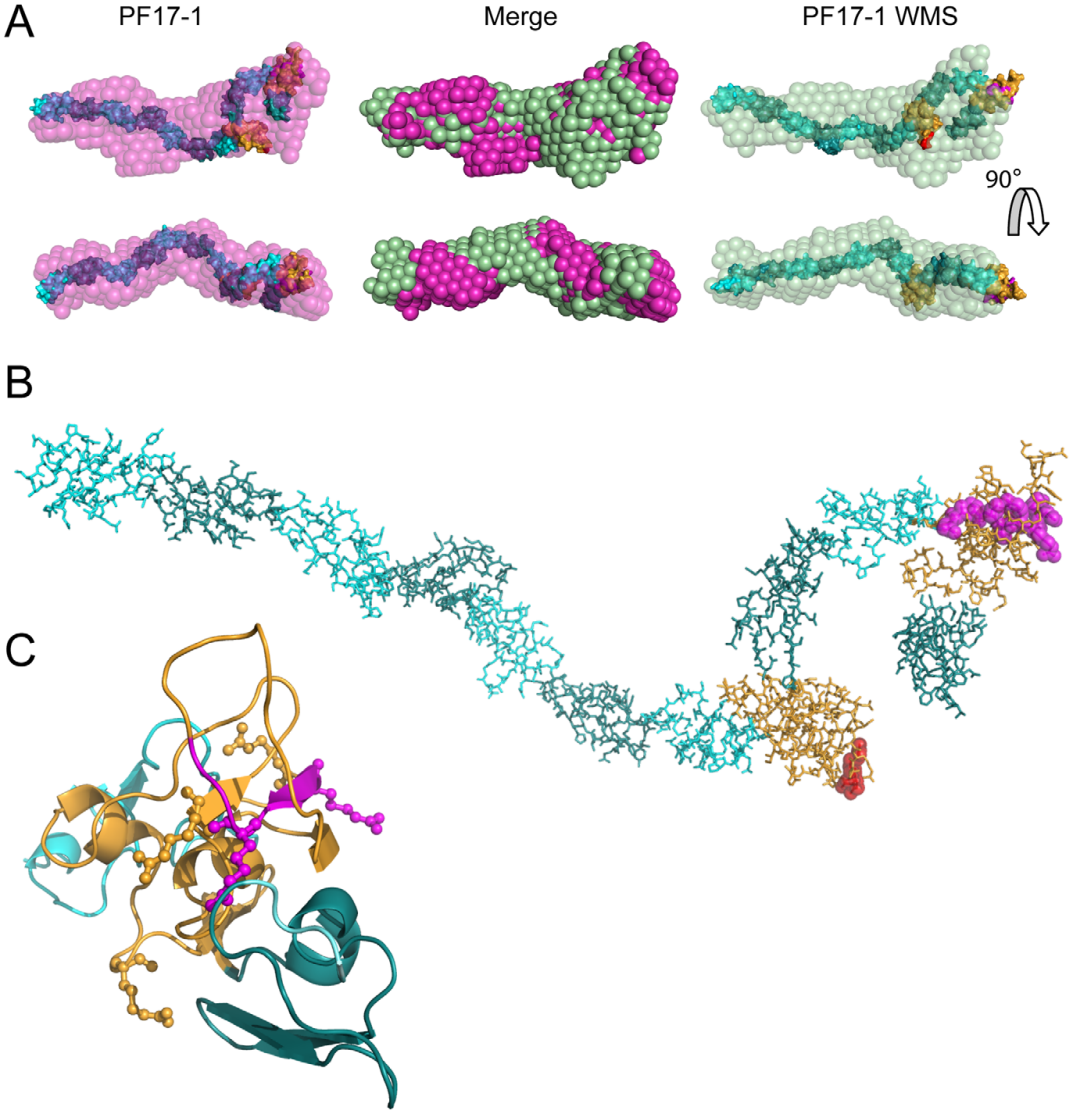
Rigid-body models of PF17-1 and PF17-1 WMS generated from SAXS analysis. ( A) Rigid Body models were generated from experimental scattering data for PF17-1 and PF17-1 WMS using SASREF. Over 20 rigid-body models were generated for each experiment and the resulting models were aligned, and a volume frequency map was generated using DAMAVER. The most probable shape from the frequency map was generated using DAMFILT, which is shown in magenta for PF17-1 and green for PF17-1 WMS, along with the rigid body model for each protein fragment that had the lowest chi squared value determined by SASREF. The overlaid/merge of the PF17-1 and PF17-1 WMS DAMFILT structures, showing their similarity are shown in the center. (B) Rigid-body model of PF17-1, showing the cbEGF domains in cyan and dark teal and TB domains in orange. The position of the RGD motif of TB4 is shown in red and the position of the 8 amino acid deletion in PF17-1 WMS is shown in magenta. (C) Model of EGF28-TB5-EGF29 of PF17-1, showing the four Arg residues involved in heparin binding as ball and sticks and the loop of 8 amino acids deleted in PF17-1WMS in magenta. The structural model was created using SwissModel, as described in Materials and Methods.

### Cell Adhesion and Spreading Assays

Attachment assays were conducted as reported [8], [9]. Briefly, 100 µl of fibrillin ligand in triplicate was added to 96-well tissue culture plates at a concentration range of 0–10 nM overnight at 4°C. Wells were blocked with the addition of 200 µl bovine serum albumin (BSA) (10 mg/ml) in phosphate-buffered saline (PBS) for 1 hour at room temperature, which was aspirated prior to the addition of 100 µl of human dermal fibroblasts (HDFs, Invitrogen) in serum free DMEM (1×10^5^ cells/ml). Cell were incubated at 37°C/5% CO_2_ for 60 minutes, and non-adherent cells were removed by aspiration followed by a single wash with 200 µl PBS. Adherent cells were fixed by the addition of 100 µl of 5% (w/v) glutaraldehyde for 30 minutes at room temperature. Wells were washed three times with water then stained with 100 µl 0.1% (w/v) crystal violet, 200 mM 2-(N-morpholino)ethanesulfonic acid (MES) solution for 1 hour at room temperature. Wells were washed a further three times with 400 µl water. Optical densities (OD) at 570 nm were measured on a Dynex MRX II microtitre plate reader with Revolution software (Dynex LabSystems, UK). Absorbance was normalized for each plate and expressed as a percentage of cell attachment to 10 nM PF17-1. All experiments were repeated at least three times.

Cell spreading was assayed by plating HDF or ARPE-19 (Adult Retinal Pigmented Epithelial) cells (ATCC) on to different protein coated surfaces. Fibrillin-1 protein fragments PF17-1, PF17-1 WMS, PF8, PF10, fibrillin-2 fragment PF17-2 or BSA were coated overnight at 200 nM onto wells of a Lab-Tek permanox 8-well chamber slides. Cells were prepared in the same way as for the cell adhesion assay. 250 µl of cells in serum-free DMEM (1.5×10^5^ cells/ml) were applied to aspirated wells and incubated at 37°C/5% CO_2_ for 120 minutes. Non-adherent cells were removed by aspiration followed by a single wash with 200 µl PBS. Cells were fixed with the addition of 3% (w/v) paraformaldehyde (200 µl) for 20 minutes, then quenched with 0.2 M glycine. Cell were permeabilized with 200 µl 0.5% Triton X-100 for 8 minutes, then blocked with 3% filtered sterilized fish skin gelatin (200 µl) for 1 hour. Focal adhesions were visualized by the addition of mouse anti-human vinculin primary antibody (1∶200) (V9131 Sigma) for 45 min followed by washing in PBS. After washing incubation with anti-mouse secondary (Alexa Fluor 488 Life Technologies) and Alexa Fluor 594 Phalloidin (#A12381 Life Technologies), both 1∶1000 dilution. The chambers were removed from the permanox slide, which was then washed in ultrapure water prior to mounting with a glass slide with Prolong Gold containing DAPI (Life Technologies). Images were collected on an Olympus BX51 upright microscope using a 60× objective and captured using a Coolsnap ES camera (Photometrics) through MetaVue Software (Molecular Devices). Specific band pass filter sets for DAPI, FITC and Texas Red were used to prevent bleed through from one channel to the next. Images were processed and analyzed using ImageJ (http://rsb.info.nih.gov/ij).

### SAXS and Rigid-body Modeling

Small angle X-ray scattering data (SAXS) for PF17-1 were collected on EMBL beamline X33 at the light source facilities DORISIII at HASYLAB/DESY [18]. Data were collected using a 30 second exposure time and 2.4 metre sample-to-detector distance to cover a momentum transfer interval 0.008<*q*<0.54 Å^−1^. The modulus of the momentum transfer is defined as q = 4Πsinθ/λ, where 2θ is the scattering angle, and λ is the wavelength. The q range was calibrated using silver behenate powder based on diffraction spacings of 58.38Å. The scattering images obtained were spherically averaged using in-house software and buffer scattering intensities subtracted using PRIMUS. Data on PF17-1 WMS were collected using standard procedures on the I22 beamline at Diamond Light Source equipped with a photon counting detector [19], [20]. The beam was focused onto the detector placed at a distance of 2 m from the sample cell and X-ray wavelength of 0.083 nm. The covered range of momentum transfer was 0.005<q<0.36 Å^−1^. The data were normalized to the intensity of the incident beam and the scattering of the buffer was subtracted using an in-house program. To check for radiation damage and aggregation during the SAXS experiment, the data were collected in 120 successive 1 s frames.

Rigid-body modeling to the experimental scattering data was performed using SASREF [21], using homology models built using the automated protein structure modeling server SwissModel [22]. EGFs 20–25 were modeled on the high resolution structural information from fibrillin-1 cbEGF12-13 (EGF16-17) [23] and EGF28-TB5-EGF29 was modeled on cbEGF22-TB4-cbEGF23 (EGF26-TB4-EGF27) [24]. 12 SASREF simulations were computed, using single domains and two EGF pairs (20–21 and 24–25). The structures generated were then superimposed using DAMAVER suite of programs (DAMAVER, DAMFILT, DAMSEL, and DAMSUP) in automatic mode [25] and compared visually for similarity. CRYSOL [26] was used for the simulation of scattering curves from the structural models, to give a measure (discrepancy factor χ) of how well the structural model fitted the experimental data, and a value of radius of gyration for each model. The hydrodynamic properties of the rigid body models were calculated using the software HYDROPRO [27] and SOMO [28] which gave a value for the radius of gyration for each rigid-body model which was compared to the experimental value from the SAXS data, and a Stokes radius and frictional ratio (*f/f*_o_) which was compared with the experimental data from analytical ultracentrifugation.

### Analytical Ultracentrifugation

Analytical ultracentrifugation (AUC) was performed in 10 mM HEPES pH 7.4, 0.1 M NaCl, 0.5 mM CaCl_2_ using a Beckman XL-A ultracentrifuge (Beckman Instruments, USA) with an An50Ti-8-hole rotor as described previously [29]. The data were interpreted with the model-based distribution of Lamm equation solutions C_(S)_ using the software Sedfit [30]. The resulting sedimentation coefficient was used to calculate Stokes (hydrodynamic) radius and frictional ratio (f/fo) using the software Sednterp [31] with molecular mass calculated from MALLS analysis.

## Results

### Heparin Binding by Fibrillin-1 and Fibrillin-2 TB5 Domains

Previously, we showed that human fibrillin-1 contains a heparin binding site in TB5, which is close to the RGD-containing TB4 domain [6]. Fibrillin-1 fragment PF17-1 was created, along with its fibrillin-2 homolog PF17-2 (Fig. 1A) in order to investigate their binding to heparin. Binding analysis was performed by comparing monomeric PF17 fragments using Surface Plasmon Resonance with a Biacore 3000 using a heparin oligosaccharide (dp20) immobilized sensor chip. PF17-2 showed a greatly decreased response level compared with PF17-1 (17.3±1.2% of PF17-1 at 800 nM) (Fig. 1D,E; Fig. S1B).

To establish whether TB5 alone mediated the interaction of PF17-1 to heparin, domain constructs were made where each TB5 domain, or the TB5 and its following cbEGF29 domain, was swapped between fibrillin-1 PF17-1 and fibrillin-2 PF17-2 (Fig. 1B). The protein fragments were analyzed by SDS-PAGE and showed the characteristic apparent increase in molecular weight on reduction (Fig. 1C). This is due to the many disulfide bonds maintaining the core protein structure intact under denaturing conditions, with only reducing conditions allowing the protein to fully denature. When the fibrillin-2 TB5 domain was placed in fibrillin-1 PF17-1 (protein fragment PF17-1 F2 T5), there was a greatly decreased response level (16.6±0.8% of PF17-1 at 800 nM), which was comparable to PF17-2. The binding was slightly improved (34.9±2.3%) when the following cbEGF from fibrillin-2 was also added (PF17-1 F2 T5E29). The inverse effect was found when the fibrillin-1 TB5 was placed in fibrillin-2 PF17-2 (PF17-2 F1 T5), since the binding level of PF17-2 was increased from 17.3±1.2% to 48.6±2.0%. The level of binding was fully restored to PF17-1 levels when the following fibrillin-1 EGF29 was added to PF17-2 (PF17-2 F1 T5E29) (98.6±7.8%) (Fig. 1D,E; Fig. S1B). Previously it has been shown that fibrillin-1 does not interact with either hyaluronan or chondroitin 6-sulfate [5], [6]. To confirm that PF17-2 also did not bind these glycosaminoglycans, fibrillin-1 PF17-1 and fibrillin-2 PF17-2 were injected over immobilized hyaluronan and chondroitin 6-sulfate at a concentration of 200 nM. There was no interaction between PF17-1 or PF17-2 fragments and these glycosaminoglycans (Fig. S1C,D), although interactions with the known hyaluronan-binding proteins, versican G1 domain and TNF-stimulated gene 6 protein (TSG-6) were confirmed.

### Heparin Binding Residues of Fibrillin-1 TB5

Previously we showed that Arg 1691, 1692, 1727 and 1752 were important for the fibrillin-1 TB5 heparin interaction [6], [9]. To see if the corresponding amino acids were present in fibrillin-2 TB5, an alignment was performed (Fig. 2A). Where different, the relevant amino acids were mutated ‘back’ to fibrillin-1 residues (Arg 1737, 1770 and 1794). A further point mutation was added to the PF17-2 K1737R fragment at amino acid 1739 Phe to Leu, in order to make the first 6 amino acids identical with fibrillin-1 TB5. The binding responses of these mutants were increased compared with wild-type PF17-2 (which had shown only 17.3±1.2% of PF17-1 binding to heparin at 800 nM; *see above*), with Arg point mutations at K1737R (44.4±1.5%), K1770R (41.9±7%) and +QI1794R (68.0±5.3%), with the third site (1794) having the greatest response (Figs. 2B; S2). The response level of the PF17-2 K1737R was further increased with the additional mutation at F1739L (63.9±3.1%). When all four sites were concurrently mutated to the fibrillin-1 sequence (PF17-2 Quad), the response level was restored to that of wild-type PF17-1 (101.8±16.9%). When the fibrillin-2 TB5 structure was modeled using the homologous fibrillin-1 crystal structure (1UZK), it was found that K1737 and F1739 were on one face of the molecule (Site 1) and K1770 and I1794 were on the opposite face (Site 2) (Fig. 2C), suggesting that the mutated PF17-2 had gained the ability to bind HS at two sites.

### PF17-1 WMS Mutant has Reduced HS Binding

Sequencing confirmed that PF17-1 WMS contained the 8 amino acid deletion found in patients displaying WMS (Fig. 3A) [14]. PF17-1 WMS purifications resolved into two peaks using gel-filtration chromatography, which corresponded to a monomer (86%) and a disulfide-linked dimer (14%) (Fig. 3B,C). By comparison, PF17-1 typically had <4% dimer present. To confirm that the separated peak samples contained only monomer or dimers, molecular weight analysis using MALLS was performed on each peak alongside wild-type PF17-1. Monomeric PF17-1 WMS had a calculated molecular weight of 77,440±1.0%, which compared well against monomeric PF17-1 (77,680±0.9%); the PF17-1 WMS dimer had a calculated molecular weight of 166,200±0.9%, which is slightly higher than expected (Fig. S3A). When monomeric PF17-1 WMS was analyzed for heparin binding, the binding response was almost completely ablated (8.7±4.3%). However, the dimeric form of this mutant binding showed some heparin binding (57.9±8.1%) (Figs. 3D, S3C). Cell adhesion to immobilized PF17-1 WMS was not significantly reduced, as expected given that syndecan-mediated focal adhesion formation is generally induced by soluble ligands such as fibronectin hepII [9], [32], [33].

### PF17-1 AD and GD Mutants have Reduced HS Binding

Six recombinant PF17-1 AD and GD point mutants (Y1696C [GD], Y1699C [GD/AD], M1714R [AD], G1726V [AD], A1728T [GD/AD], S1750R [AD]) were purified (Fig. 4A, S4A), and sequencing confirmed that each contained the appropriate mutation (not shown). Purification of these mutants revealed that all were mainly monomeric, like wild-type PF17-1 (not shown). Using monomers that were further purified by size fractionation and verified by MALLS (not shown), the AD and GD mutant PF17-1 fragments were analyzed for heparin binding, as above. The binding responses showed that binding of 5 of the mutants to heparin was significantly reduced compared to wild-type PF17-1. The sixth mutant, S1750R, also appeared to have reduced heparin binding, but this result was not statistically significant (Fig. 4B, S4B). The position of the S1750R mutant of the structure of fibrillin-1 TB5 is set apart from the other mutations, which are clustered around the WMS loop (Fig. 4C).

### Reduced Cell Adhesion to PF17-2 is not Rescued by PF17-1 TB5

PF17-1 and PF17-2 both contain the integrin binding motif (RGD) located in TB4. Wepreviously identified a integrin synergy region upstream of fibrillin-1 TB4 that is required for robust α5β1 engagement [9]. To determine whether the fibrillin-2 PF17-2 fragment had comparable cell attachment properties to fibrillin-1 PF17-1, we conducted cell adhesion assays using PF17-1 and PF17-2, in HDFs (Figs. 5, S5). HDFs attached to PF17-2 at a significantly lower level (73.8±6.9%) than to PF17-2. We also tested whether a domain swap that restores heparin binding in PF17-2 (PF17-2 F1 T5E29, with PF17-1 F2 T5E29 control) (*see* Figs. 1,2) also enhanced cell attachment to PF17-2 (Fig. 5). However, the presence of the fibrillin-1 heparin binding sequence (PF17-2 F1 T5E29) did not significantly increase cell attachment across the coating concentration range. Thus, the reduced cell attachment capacity of PF17-2 cannot be due to reduced heparin/HS binding.

### Cell Spreading of Monomers is Mainly Driven by RGD-containing TB4

Cell spreading and focal adhesion formation was assessed by plating cells onto tissue-culture plastic surfaces (permanox) coated with fibrillin-1 protein fragments PF17-1, PF17-1 WMS, FBN1 PF8 (which the contains the RGD motif containing TB4 but not TB5) and FBN1 PF10 (which contains heparin binding site TB5 but not TB4) (Fig. S6A). Fibrillin-2 fragment PF17-2 and BSA were also used. ARPE-19 cells were found to spread and form vinculin-containing focal adhesions equally well on the protein fragments containing the RGD motif; PF17-1, PF17-2, PF17-1 WMS and PF1 PF8. No significant difference could be seen in cell spreading or actin cyto-skeleton formation between the fragments that bound heparin well (PF17-1), poorly (PF17-2 and PF17-1 WMS) or not at all FBN1 PF8 (Figs. S6B, S6C). ARPE-19 cells were found to spread poorly on the fragment FBN PF10, although there was significantly more spreading than the cells plated on BSA (Fig. S6D). Virtually identical results were found with the spreading of HDF cells (data not shown). In these experiments, whilst validated monomers enabled comparison between fragments, physiologically fibrillin-1 occurs as a multimer (microfibril) which is likely to have much stronger heparin sulfate interactivity.

### SAXS and AUC Analysis of PF17-1 and PF17-1 WMS

We previously showed that subfragments of PF17-1 are not linear in physiological solution [34]. Here we determined the solution structure of PF17-1 and the domain swap mutants, to determine the organization of this critical region of fibrillin-1 and to check whether these mutations introduced any gross structural changes (Figs. 6, S7). Twenty rigid body models were generated for PF17-1 SAXS data using SASREF [21] which gave an average fit to the experimental scattering curve value (χ^2^) of 1.41±0.04, and 29 rigid-body models were generated for PF17-1 WMS (χ^2^ = 0.66±0.01). All models were aligned and an average structural envelope and frequency map was generated with DAMAVER suite of programs. The most probable structural envelope for PF17-1 and PF17-1 WMS was calculated using DAMFILT (Fig. 5A, Figure S5). Both PF17-1 and PF17-1 WMS gave very similar DAMFILT structures, with the majority of the rigid-body models predicted from both SAXS data having a largely linear sequence of cbEGF domains at the N terminal (EGF20-26), with a looped structure at the C-terminal end which contained the two TB domains (TB4 and TB5) (Fig. 6B). This alignment also matched previous structural studies on fragments consisting of the N and C terminal regions of PF17 (PF8 and PF9) [34]. Theoretical radii of gyration were determined for each rigid-body model, using CRYSOL, which does a statistical comparison of the rigid-body model with the experimental SAXS data (CRYSOL discrepancy value PF17-1 1.41±0.05, and PF17-1 WMS 0.66±0.01). This analysis gave similar average values of 6.34±0.02 nm for PF17-1 and 6.15±0.06 nm for PF17-1 WMS. Bead modeling of rigid-body models, using HYDROPRO and SOMO, also gave similar radii of gyration of 6.31±0.02 and 6.32±0.06 nm respectively for PF17-1 and 6.16±0.06 and 6.14±0.06 nm for PF17-1 WMS (Table 1). Analytical ultracentrifugation was performed on both protein fragments and the hydrodynamic (Stokes) radii were computed. Both proteins had almost identical sedimentation coefficients (S_20,w_) of 3.68 and 3.65 Svedberg (Fig. S3B) for PF17-1 and PF17-1 WMS respectively, which gave calculated hydrodynamic radii of 5.04 and 5.06 nM respectively. The frictional ration (*f/f*_o_) was calculated to be 1.79 for both proteins. These values compared well with the calculated hydrodynamic radii and frictional ratios calculated from the rigid-body models using bead modeling (Table 1). In summary, PF17-1 was not linear in physiological solution, and none of the mutants tested affected its organization, as judged by these methods.

**Table 1:** Calculated radii of gyration and hydrodynamic radii of PF17-1 and PF17-1 WMS.

	PF17-1		PF17-1WMS	
	Hydrodynamic radius(nm)	Radius of gyration(nm)	Hydrodynamic radiusnm)	Radius of gyration(nm)
SAXS (exp)		5.8		5.9
CRYSOL (theo)		6.343±0.023		6.154±0.06
HYDROPRO (theo)	5.08±0.02	6.31±0.02	4.98±0.02	6.16±0.06
SOMO (theo)	4.76±0.05	6.32±0.06	4.66±0.02	6.14±0.06
AUC (exp)	5.04		5.06	
	Frictional ratio (f/fo)		Frictional ratio (f/fo)	
HYDROPRO (theo)	1.81±0.01		1.84±0.01	
AUC (exp)	1.79		1.79	

Table showing the calculated values for hydrodynamic radius, radius of gyration, and frictional ratios for protein fragments PF17-1 and PF17-1 WMS. Values from experimental results (exp) were calculated from SAXS data (using Guinier approximation) and analytical ultracentrifugation data (AUC) (using Sednterp). Theoretical (theo) values for the hydrodynamic radius and radius of gyration were calculated from rigid-body models generated from SAXS data using SASREF, using the programs CRYSOL, HYDROPRO and SOMO. Theoretical frictional ratio values were also calculated from rigid –body models using HYDROPRO.

## Discussion

It is clear that fibrillin-1 is one of the major HS-binding molecules of the extracellular matrix, with multiple HS binding sites that have key roles in microfibril assembly and cellular interactions [4]–[7]. Here we focused on HS binding to the TB5 domain, which we have previously shown to contribute to integrin-mediated cellular interactions in a manner analogous with the fibronectin hepII region [9]. Using fibrillin fragments encompassing both RGD-containing TB4 and downstream HS-binding TB5, we discovered that, unlike fibrillin-1, HS binding to the fibrillin-2 TB5 domain is very weak. This novel functional difference between the two fibrillin isoforms enabled us both to map fibrillin-1 HS binding to two sites on opposite sides of TB5 and to explore HS binding by TB5 mutations causing WMS, AD and GD (non-cysteine in WT) mutants, using a site-directed mutagenesis approach. These disease-causing mutations all mapped to the two HS identified binding sites, and the mutants had reduced HS binding, implying a contribution to these disease pathologies.

HS binding to fibrillin-1 is known to be greatly enhanced by fibrillin-1 multimerization [4]–[7]. In our first study of HS binding to fibrillin-1, we showed that fragments PF10 and PF11 which both contain TB5 and had been purified only by nickel affinity columns, had high affinity for heparin/HS [6]. Here, we prepared and validated monomers of all fragments specifically in order that we could directly compare all wild-type and mutant fragments. A domain swap strategy, exploiting weakly-binding PF17-2 and strongly binding PF17-1, was used to confirm that the HS-binding site in this region of fibrillin-1 was indeed within TB5. Interestingly, full heparin-binding capacity could only be restored to fibrillin-2 TB5 when fibrillin-1 TB5 and the following EGF29 were both swapped, probably reflecting the precise structural linkage of TB5 and EGF29 [24]. The PF17-2 fragment had poor cell adhesion properties that were not restored by the fibrillin-1 TB5 domain and EGF29, indicating that the fibrillin-2 RGD region is not as efficient at mediating cell attachment as that of fibrillin-1. However, syndecan-mediated focal adhesion formation is generally induced by soluble ligands such as fibronectin hepII [9], [32], [33]. We speculate that TB5 may trigger similar signaling events *in vivo* to modulate integrin function and cell behavior [9]. Although cell attachment was poor with the PF17-2 fragment, cell spreading of monomers of PF17-2, PF17-1 WMS and the only TB4 containing fragment FBN1 PF8 was indistinguishable to PF17-1, and was therefore largely dependent on the RGD motif, not the heparin binding motif. However there was some significant spreading with the TB5 only containing fragment FBN1 PF10 indicating that the heparin binding site does interact with the cell surface. Physiologically fibrillin-1 multimers may bind more strongly to heparin sulfate, as seen in vitro [5].

It remains unclear why the vast majority of known mutations throughout fibrillin-1 cause Marfan syndrome, but that certain mutations in TB5 specifically cause WMS, AD and GD [16] and in the RGD-containing TB4 cause Stiff Skin syndrome, a congenital scleroderma with thickened skin and limited joint mobility [35]. Here we sought new insights into the molecular pathogenesis of WMS, AD and GD by comparing the structure and HS-binding function of wild-type and disease-causing mutant fibrillin-1 cell adhesion PF17-1 fragments. Having mapped the two HS binding sites with fibrillin1 PF17-1 region, it was striking that the WMS deletion mutation, and the six of the non-cysteine AD and GD point mutations within TB5 all mapped to these two regions (Fig. 4C). We did not test two other non-cysteine mutations which fall in the TB5-EGF29 linker region that we have here shown to be important for optimal HS binding. We found that the PF17-1 WMS mutant and five of the PF17-1 AD and GD mutants showed significantly reduced HS binding; the sixth mutant also showed reduced HS binding although not statistically significant. The WMS mutant fragment was 86% monomeric, although low level of disulfide-bonded dimers with HS binding potential were detected which may result from the free cysteine residue caused by deletion a Cys 1695. However, none of the AD or GD mutants had an unpaired cysteine and all were predominantly monomeric like the wild-type protein. Thus, these mutations in the TB5 HS binding sites clearly disrupt HS binding which, in the physiological multimeric microfibril context, will have a marked effect on cellular interactions.

SAXS analysis of PF17-1 monomeric fragments in solution revealed, as previously reported using shorter fragments [34], that this region of the molecule is not linear. Both wild-type PF17-1 and PF17-1 WMS fragments have virtually the same structural envelopes from SAXS and AUC data, so the deletion mutation does not grossly alter molecular structure within the limits of the resolution of the techniques. The defects caused by this WMS mutation may instead reflect defective fibrillin-1 interactions with HS, as demonstrated here at the molecular level.

Whilst autosomal dominant WMS is caused by a deletion in fibrillin-1 TB5, autosomal recessive WMS is caused by mutations in metalloproteinases ADAMTS10 [36] and ADAMTS17 [37]. Recently it was shown that ADAMTS10 can bind fibrillin-1, co-localize with microfibrils and enhance their deposition [38]. Although it is not known how ADAMTS molecules enhance microfibril deposition, defects in this process may explain how ADAMTS mutations cause WMS. Our study, which demonstrates that a WMS-causing fibrillin-1 mutation disrupts HS binding, leads us to speculate that pericellular HS interactions may somehow be important for ADAMTS-mediated microfibril deposition since ADAMTS molecules can bind HS and associate with syndecans [39].

In summary, we have shown that fibrillin-2 TB5 binds HS weakly compared to fibrillin-1, mapped two heparin binding sites on fibrillin-1 TB5, and shown that disease-causing WMS, AD and GD mutations all map to, and disrupt heparin binding. Our study thus sheds light on the structure and function of this important cell-interacting region of fibrillin-1 and contributes to our understanding of the pathogenesis of diseases caused by mutations in this domain.

## Supporting Information

Figure S1 **Biacore analysis of binding to fibrillin-1 fragment PF17-1, fibrillin-2 fragment PF17-2 and PF17 domain swaps.** (A) Schematic of construction of domain swap fragments using overlap PCR. In the 1^st^ round of PCR, 3 separate fragments are generated that have overlapping sequences using 3 separate primer pairs (indicated). In the 2^nd^ round of PCR, the 3 fragments are combined with fragment start and end primers to give the end product. (B) Fibrillin-1 and fibrillin-2 protein fragments were injected over the over the heparin-oligosaccharide-immobilized surface at concentrations ranging from 0 to 800 nM. One typical response curve is shown for each interaction, showing response difference (Resp. Diff.) plotted against time. Each experiment was repeated three times. (C) Response curves of PF17-1 and PF17-2 injected over immobilized hyaluronan. Also injected were known hyaluronan binding proteins; G1 domain of versican (Versican G1) and TNF-stimulated gene 6 protein (TSG6). All proteins were at a concentration of 200 nM. (D) Response curves of 200 nM PF17-1 and PF17-2 injected over immobilized chondroitin-6-sulphate.
(TIF)

Figure S2 **Biacore analysis of binding to fibrillin-1 fragment PF17-1, fibrillin-2 fragment PF17-2 and PF17-2 point mutations.** (A) Typical response curve of fibrillin-1 and fibrillin-2 protein fragments injected over the heparin-oligosaccharide-immobilized surface, as described in Figure S1 legend. (B) The average response difference of the three experiments was plotted against concentration (nM). It was shown that the binding response was increased for both heparin binding site 1 (PF17-2 K1737R and PF17-2 K1737R/F1739L) and heparin binding site 2 (PF17-2 K1770R, PF17-2+QI1794R) point mutations. The binding response was fully restored to PF17-1 levels when both heparin binding sites were restored (PF17-2 Quad). (C) SDS-PAGE analysis of PF17-1, PF17-2 and PF17-2 mutants indicated by the roman numeral shown in (B), run under non-reducing (NR) and reducing conditions (R) using a 4–12% RunBlue gel (Expedeon UK).
(TIF)

Figure S3 **MALLS, Biacore and AUC analysis of PF17-1 and PF17-1 WMS.** (A) Multi-Angle Laser Light Scattering analysis of PF17-1, PF17-1 WMS Monomer (86%), and PF17-1 WMS Dimer (14%), showing the light scattering (LS) trace of detector 11 (90 degrees) and the differential refractive index(diff. RI). Shown is the calculated molecular weight of the peaks indicated by the peak boundaries. (B) Distribution plot using continuous c(s) sedimentation model of Sedfit generated by AUC. Also shown is the sedimentation values (S) calculated by integration of the two respective peaks. (C) Biacore analysis of heparin binding to fibrillin-1 fragment PF17-1, PF17-1 WMS monomer and dimer. FBN1 protein fragments were injected over the heparin-oligosaccharide-immobilized surface at concentrations ranging from 0 to 800 nM. One typical response curve is shown for each interaction, showing response difference (Resp. Diff.) plotted against time. Each experiment was repeated three times.
(TIF)

Figure S4 **BIAcore and SDS-PAGE analysis of fibrillin-1 fragment PF17-1, GD/AD point mutations.** (A) SDS-PAGE analysis of PF17-1, with GD/AD mutants run under non-reducing (NR) conditions using a 4–12% RunBlue gel (Expedeon UK). (B) FBN1 protein fragments were injected over the heparin-oligosaccharide-immobilized surface at concentrations ranging from 0 to 400 nM. The average response difference of the two experiments was plotted against concentration (nM).
(TIF)

Figure S5 **Cell attachment of human dermal fibroblasts the fibrillin-1 and fibrillin-2 protein fragments.** Cell attachment of human dermal fibroblasts (HDF) to PF17-1, PF17-2, PF17-1 F2 T5E29, PF17-2 F1 T5E29 and PF17-1 WMS. HDF were added to cell culture plate wells pre-incubated with increasing concentrations of protein fragments (0–10 nM), for 1 hour. After removal of non-adhered cells, adhered cells were stained with crystal violet and the optical densities (OD) at 570 nm were measured as described in Materials and Methods. Values were normalized to the percentage of cell attachment of PF17-1 at 10 nM. Also shown are the statistical significances of the difference to PF17-1 where P value <0.05, *; <0.01, **.
(TIF)

Figure S6 **Cell spreading of adult retinal pigmented epithelial cells (ARPE-19) on fibrillin-1 and fibrillin-2 protein fragments.** (A) Protein fragments used in cells spreading assay. Domain nomenclature is as shown in Fig.1. Protein fragments FBN1 PF8 and FBN1 PF10 were also used to assess the cell spreading properties of the RGD motif containing domain TB4, and the heparin binding domain TB5, each separately. (B) ARPE-19 cells were allowed to adhere on protein-coated permanox surface (indicated) for 2 hours prior to fixation and staining, as described in the Materials and Methods. Cells were stained with anti-vinculin antibody (green), the actin cytoskeleton was detected with Alexa Fluor 594 Phalloidin (red), and the nucleus with DAPI (blue). (C) Visualisation of the actin cyto-skeleton utilized Alexa Fluor 594 Phalloidin only. (D) (i) Multiple fields of view of ARPE-19 cells adherent to BSA and FBN1 PF10 as described in panel A. (ii) Box and whiskers plot of the average cell diameter of adhered cells on BSA and PF10. Bars represent the range of values; bottom of the box 25^th^ percentile, middle line the mean and top of the box the 75^th^ percentile. Also shown are the statistical significance of the difference to BSA where P value <0.001, ***.
(TIF)

Figure S7 **Rigid-body modeling of PF17-1 and PF17-1 WMS SAXS data.** (A) Rigid-body models of PF17-1 and PF17-1 WMS were generated using SASREF using structural models of EGF20-21, EGF22, EGF23, EGF24-25, EGF26, TB4, EGF27, EGF28, TB5 and EGF29, as colored in schematic diagram of PF17. Shown are the best fitting models for PF17-1 and PF17-1 WMS, along with the most probable shape of the aligned models using DAMAVER and DAMFILT. Also shown are the 3 next best fitting models for each protein with their SASREF fitting error values. (B) Plots showing the experimental SAXS data plotted in blue as a function of q compared with the theoretical fit of the modeled structure with SASREF, shown in red.
(TIF)

Table S1 List of oligonucleotide primers used in this study. List of primers used for domain swap constructs and site directed mutagenesis constructs. Restriction sites are shown italic. Domain overlap primers show amino acids encoded by the primer sequence.
(DOCX)
