# Supplementary figures and images for: Fibrillin-1 Mutations Causing Weill-Marchesani Syndrome and Acromicric and Geleophysic Dysplasias Disrupt Heparan Sulfate Interactions
Source: PLoS One. 2012 Nov 2;7(11):e48634. doi: 10.1371/journal.pone.0048634 (PMC3487758; doi:10.1371/journal.pone.0048634)

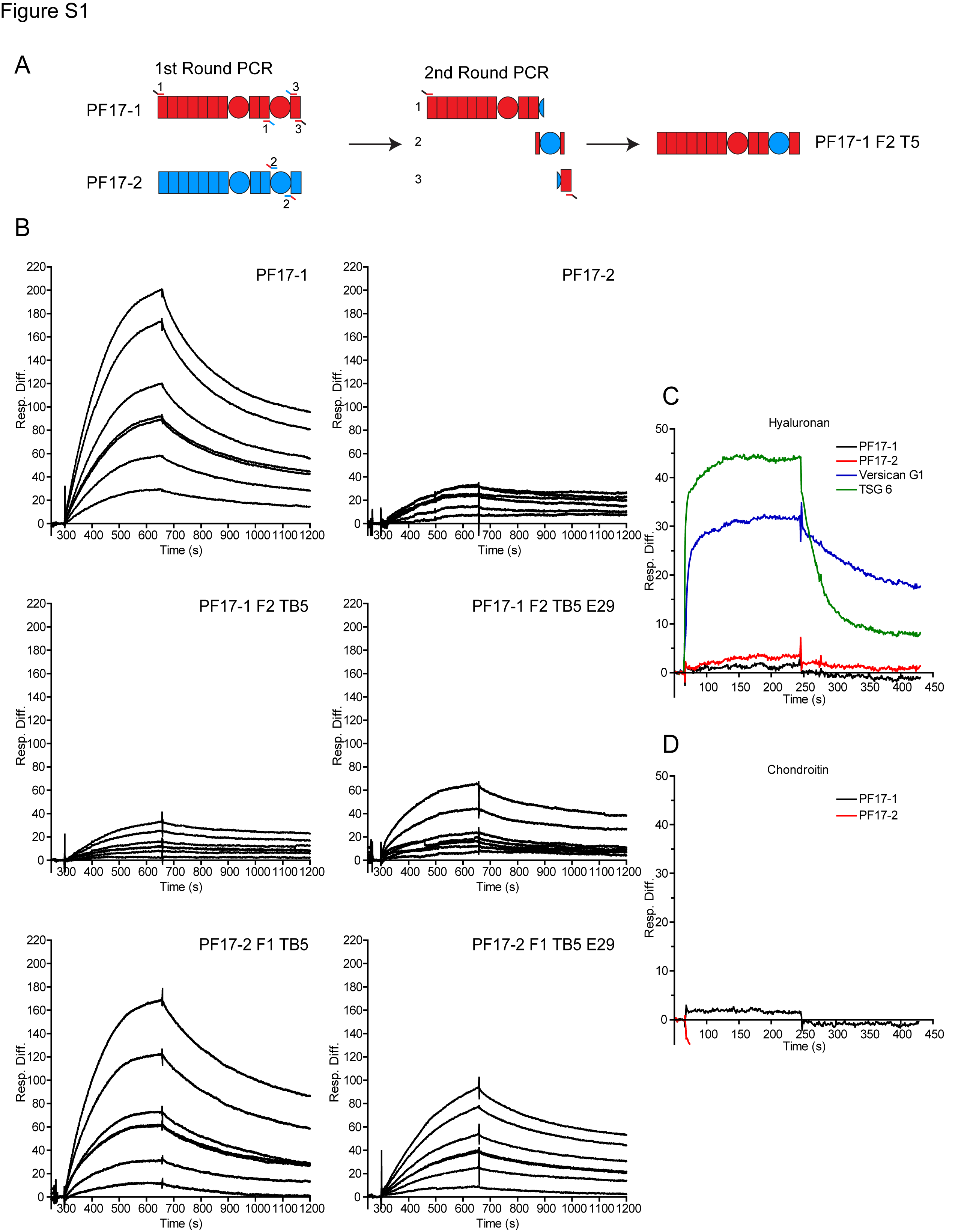

Supplement: Figure S1 — Biacore analysis of binding to fibrillin-1 fragment PF17-1, fibrillin-2 fragment PF17-2 and PF17 domain swaps. (A) Schematic of construction of domain swap fragments using overlap PCR. In the 1st round of PCR, 3 separate fragments are generated that have overlapping sequences using 3 separate primer pairs (indicated). In the 2nd round of PCR, the 3 fragments are combined with fragment start and end primers to give the end product. (B) Fibrillin-1 and fibrillin-2 protein fragments were injected over the over the heparin-oligosaccharide-immobilized surface at concentrations ranging from 0 to 800 nM. One typical response curve is shown for each interaction, showing response difference (Resp. Diff.) plotted against time. Each experiment was repeated three times. (C) Response curves of PF17-1 and PF17-2 injected over immobilized hyaluronan. Also injected were known hyaluronan binding proteins; G1 domain of versican (Versican G1) and TNF-stimulated gene 6 protein (TSG6). All proteins were at a concentration of 200 nM. (D) Response curves of 200 nM PF17-1 and PF17-2 injected over immobilized chondroitin-6-sulphate. (TIF) [file pone.0048634.s001.tif]

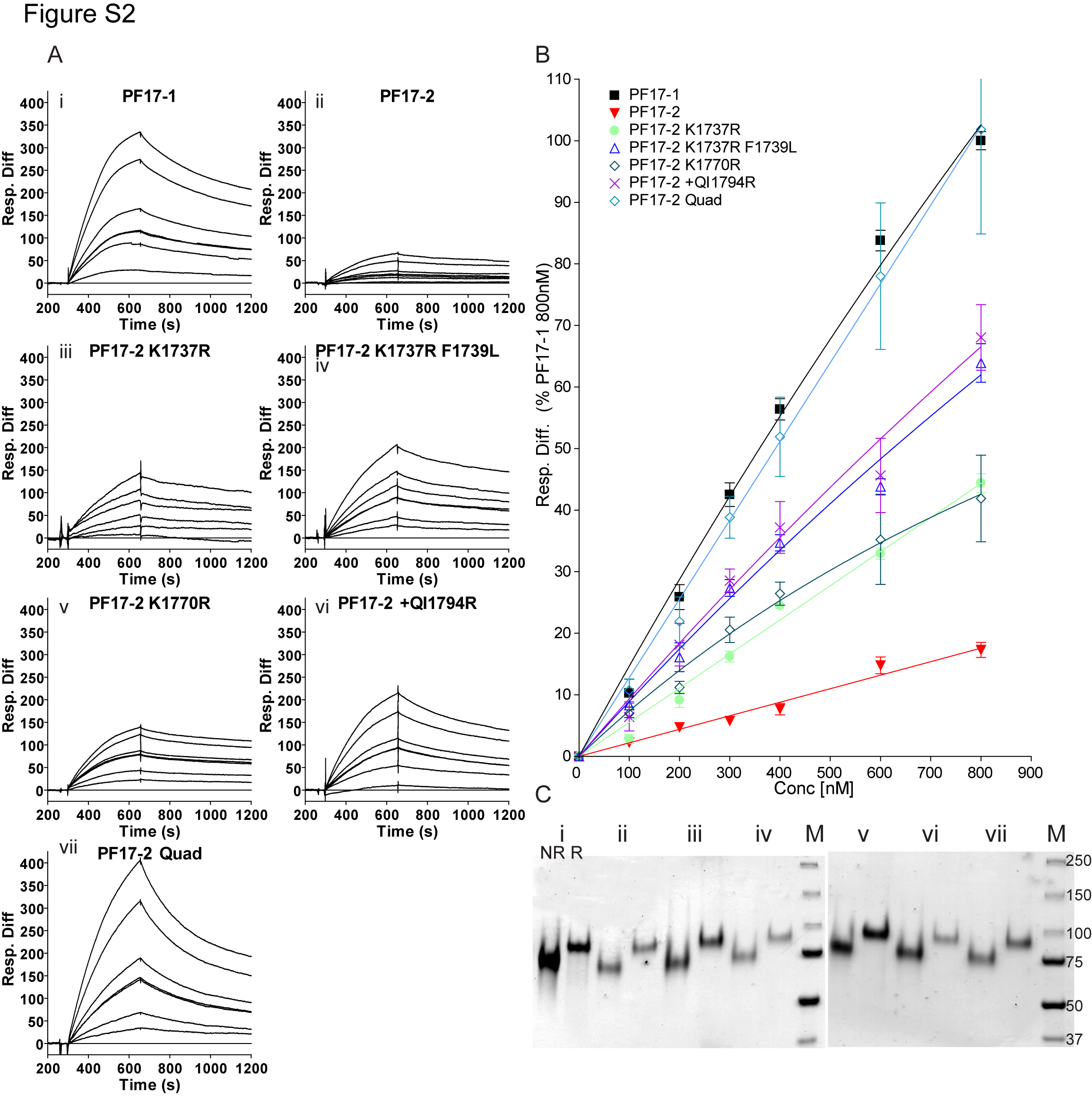

Supplement: Figure S2 — Biacore analysis of binding to fibrillin-1 fragment PF17-1, fibrillin-2 fragment PF17-2 and PF17-2 point mutations. (A) Typical response curve of fibrillin-1 and fibrillin-2 protein fragments injected over the heparin-oligosaccharide-immobilized surface, as described in Figure S1 legend. (B) The average response difference of the three experiments was plotted against concentration (nM). It was shown that the binding response was increased for both heparin binding site 1 (PF17-2 K1737R and PF17-2 K1737R/F1739L) and heparin binding site 2 (PF17-2 K1770R, PF17-2+QI1794R) point mutations. The binding response was fully restored to PF17-1 levels when both heparin binding sites were restored (PF17-2 Quad). (C) SDS-PAGE analysis of PF17-1, PF17-2 and PF17-2 mutants indicated by the roman numeral shown in (B), run under non-reducing (NR) and reducing conditions (R) using a 4–12% RunBlue gel (Expedeon UK). (TIF) [file pone.0048634.s002.tif]

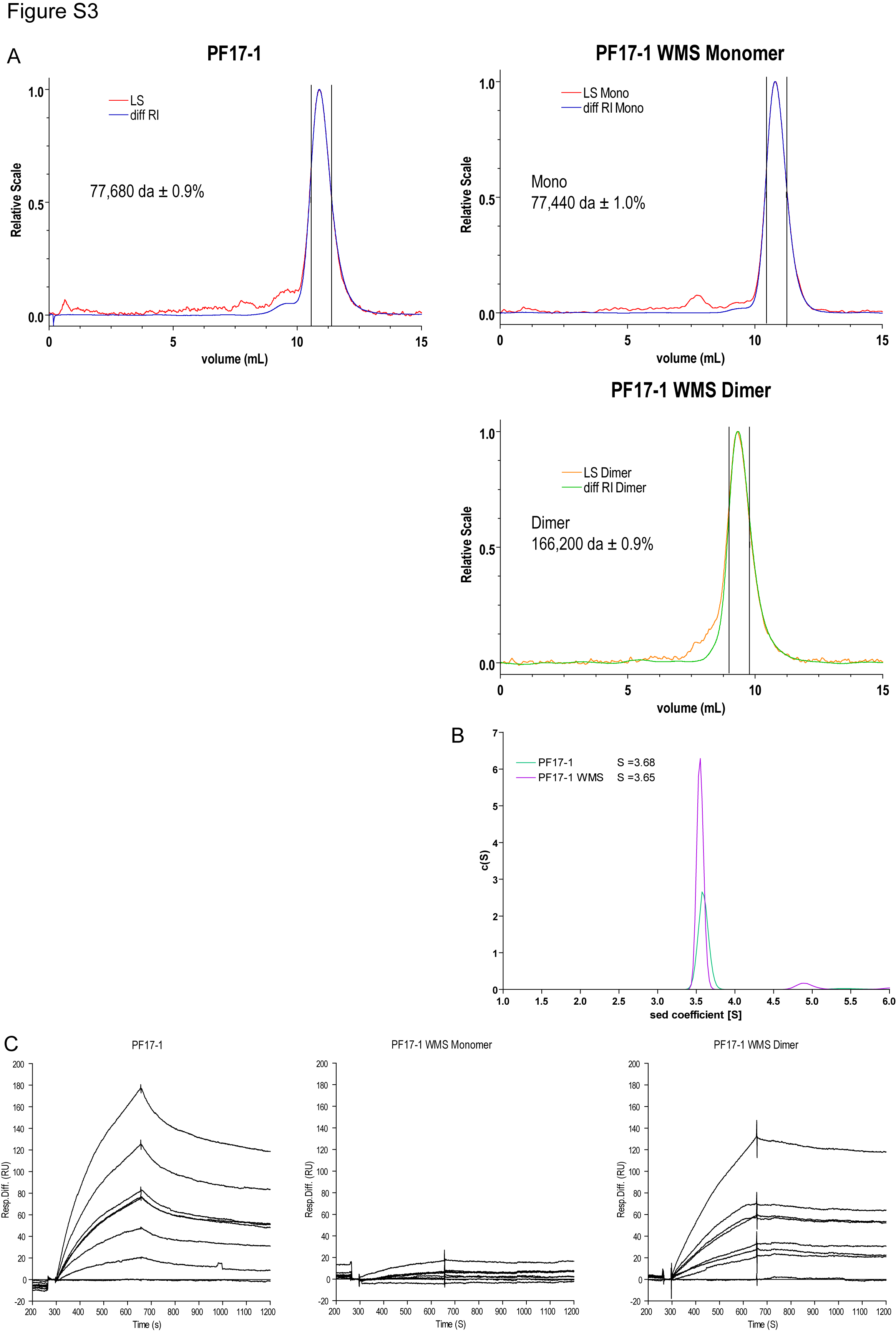

Supplement: Figure S3 — MALLS, Biacore and AUC analysis of PF17-1 and PF17-1 WMS. (A) Multi-Angle Laser Light Scattering analysis of PF17-1, PF17-1 WMS Monomer (86%), and PF17-1 WMS Dimer (14%), showing the light scattering (LS) trace of detector 11 (90 degrees) and the differential refractive index(diff. RI). Shown is the calculated molecular weight of the peaks indicated by the peak boundaries. (B) Distribution plot using continuous c(s) sedimentation model of Sedfit generated by AUC. Also shown is the sedimentation values (S) calculated by integration of the two respective peaks. (C) Biacore analysis of heparin binding to fibrillin-1 fragment PF17-1, PF17-1 WMS monomer and dimer. FBN1 protein fragments were injected over the heparin-oligosaccharide-immobilized surface at concentrations ranging from 0 to 800 nM. One typical response curve is shown for each interaction, showing response difference (Resp. Diff.) plotted against time. Each experiment was repeated three times. (TIF) [file pone.0048634.s003.tif]

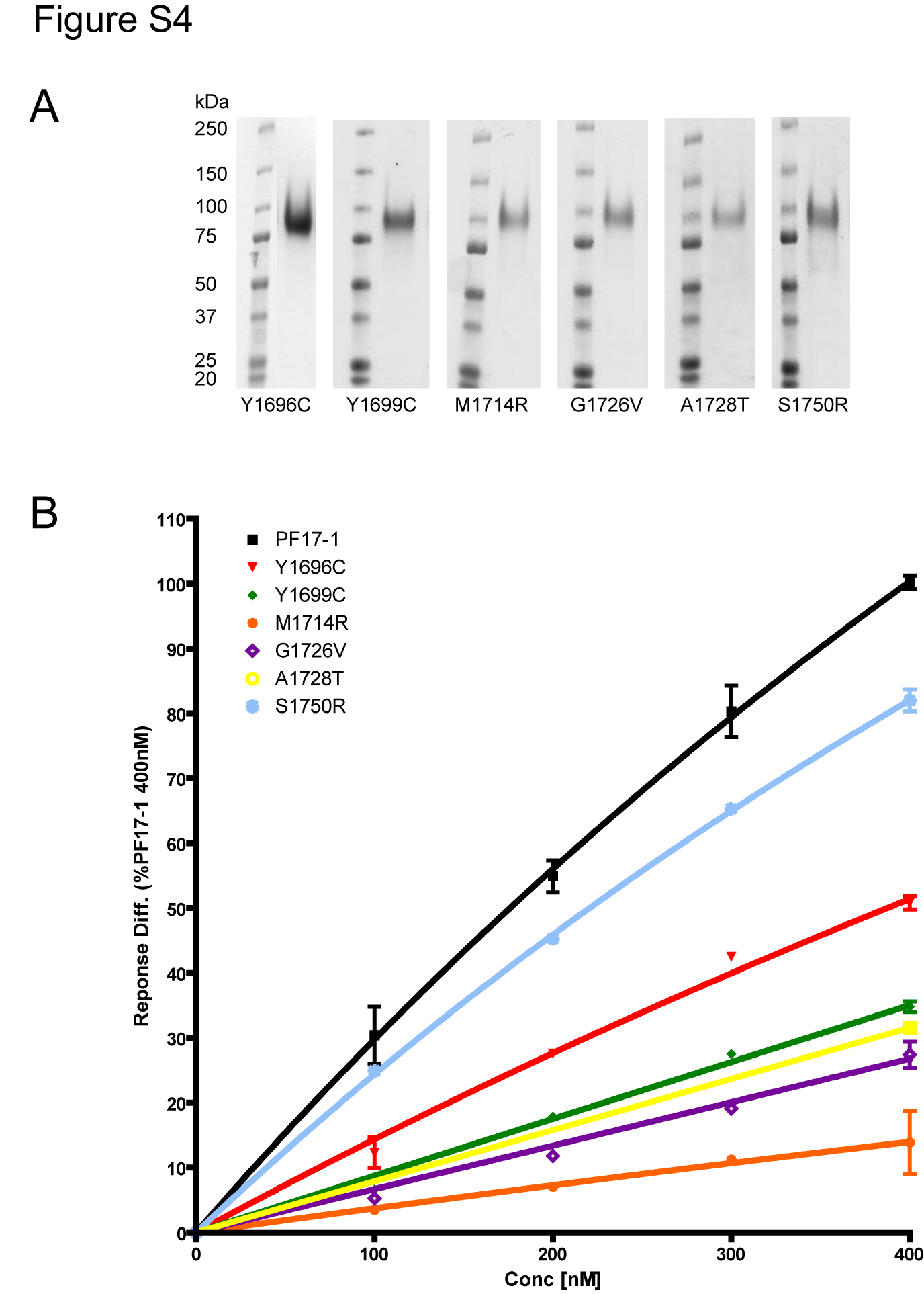

Supplement: Figure S4 — BIAcore and SDS-PAGE analysis of fibrillin-1 fragment PF17-1, GD/AD point mutations. (A) SDS-PAGE analysis of PF17-1, with GD/AD mutants run under non-reducing (NR) conditions using a 4–12% RunBlue gel (Expedeon UK). (B) FBN1 protein fragments were injected over the heparin-oligosaccharide-immobilized surface at concentrations ranging from 0 to 400 nM. The average response difference of the two experiments was plotted against concentration (nM). (TIF) [file pone.0048634.s004.tif]

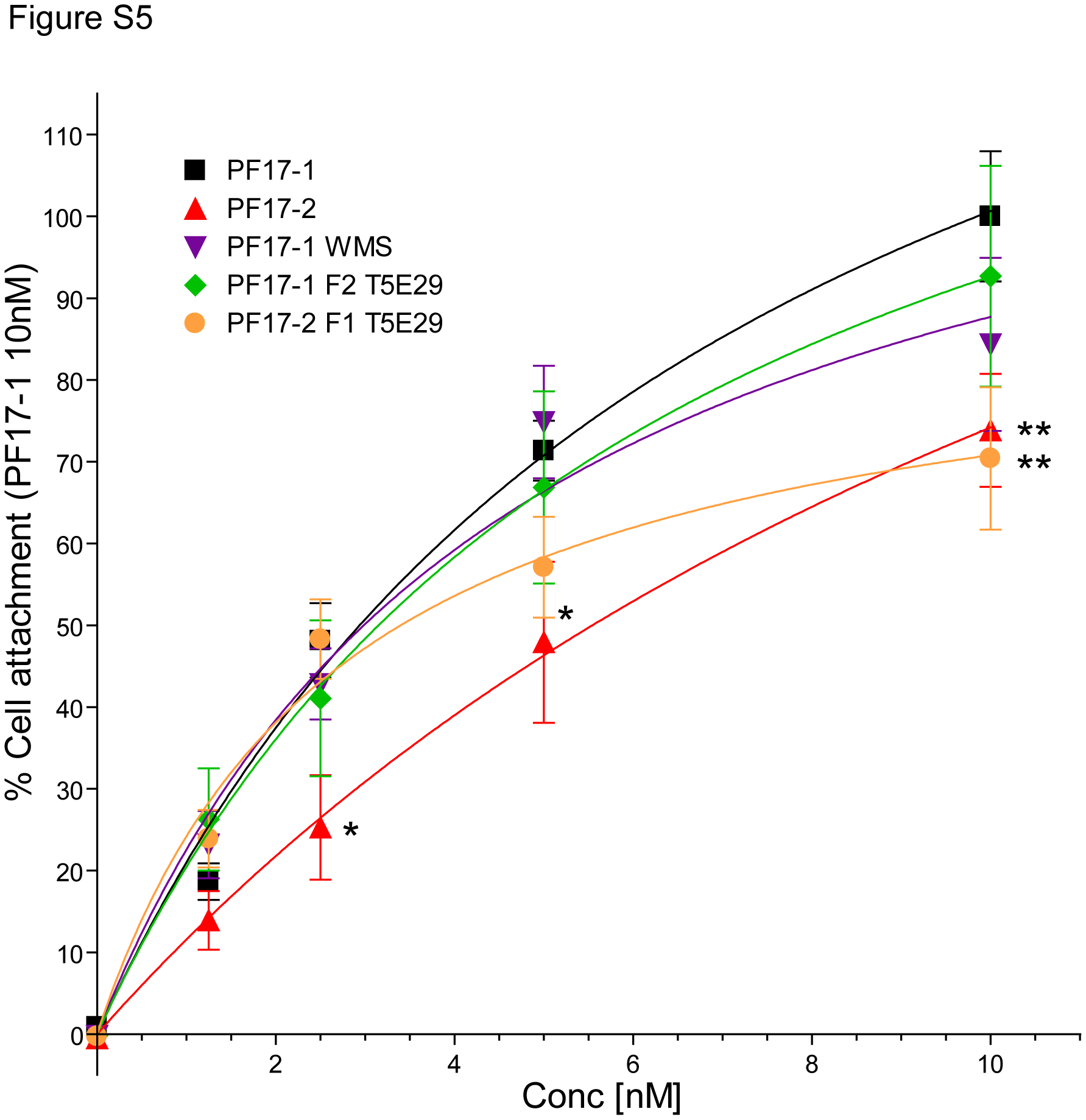

Supplement: Figure S5 — Cell attachment of human dermal fibroblasts the fibrillin-1 and fibrillin-2 protein fragments. Cell attachment of human dermal fibroblasts (HDF) to PF17-1, PF17-2, PF17-1 F2 T5E29, PF17-2 F1 T5E29 and PF17-1 WMS. HDF were added to cell culture plate wells pre-incubated with increasing concentrations of protein fragments (0–10 nM), for 1 hour. After removal of non-adhered cells, adhered cells were stained with crystal violet and the optical densities (OD) at 570 nm were measured as described in Materials and Methods. Values were normalized to the percentage of cell attachment of PF17-1 at 10 nM. Also shown are the statistical significances of the difference to PF17-1 where P value <0.05, *; <0.01, **. (TIF) [file pone.0048634.s005.tif]

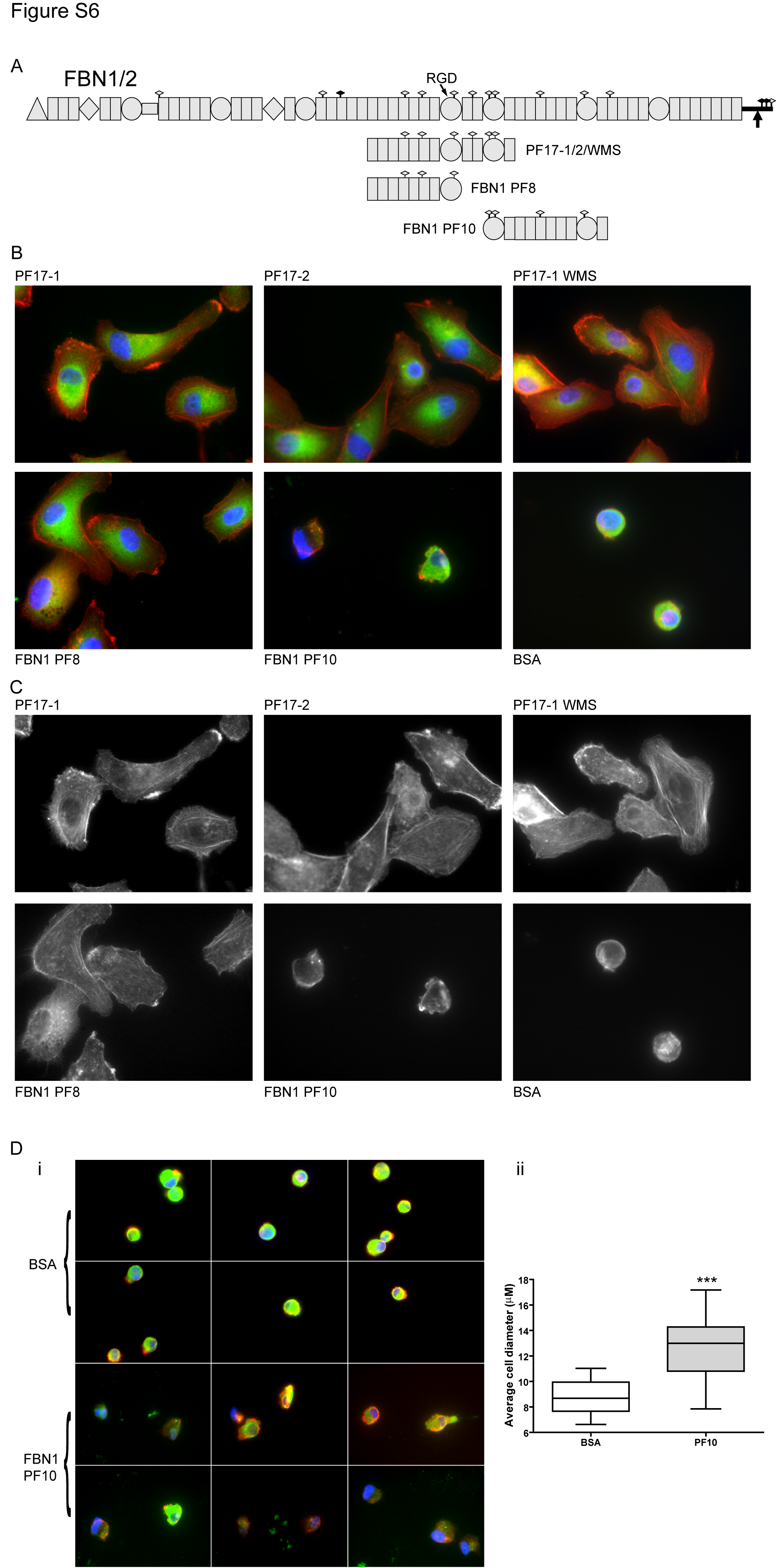

Supplement: Figure S6 — Cell spreading of adult retinal pigmented epithelial cells (ARPE-19) on fibrillin-1 and fibrillin-2 protein fragments. (A) Protein fragments used in cells spreading assay. Domain nomenclature is as shown in Fig.1. Protein fragments FBN1 PF8 and FBN1 PF10 were also used to assess the cell spreading properties of the RGD motif containing domain TB4, and the heparin binding domain TB5, each separately. (B) ARPE-19 cells were allowed to adhere on protein-coated permanox surface (indicated) for 2 hours prior to fixation and staining, as described in the Materials and Methods. Cells were stained with anti-vinculin antibody (green), the actin cytoskeleton was detected with Alexa Fluor 594 Phalloidin (red), and the nucleus with DAPI (blue). (C) Visualisation of the actin cyto-skeleton utilized Alexa Fluor 594 Phalloidin only. (D) (i) Multiple fields of view of ARPE-19 cells adherent to BSA and FBN1 PF10 as described in panel A. (ii) Box and whiskers plot of the average cell diameter of adhered cells on BSA and PF10. Bars represent the range of values; bottom of the box 25th percentile, middle line the mean and top of the box the 75th percentile. Also shown are the statistical significance of the difference to BSA where P value <0.001, ***. (TIF) [file pone.0048634.s006.tif]

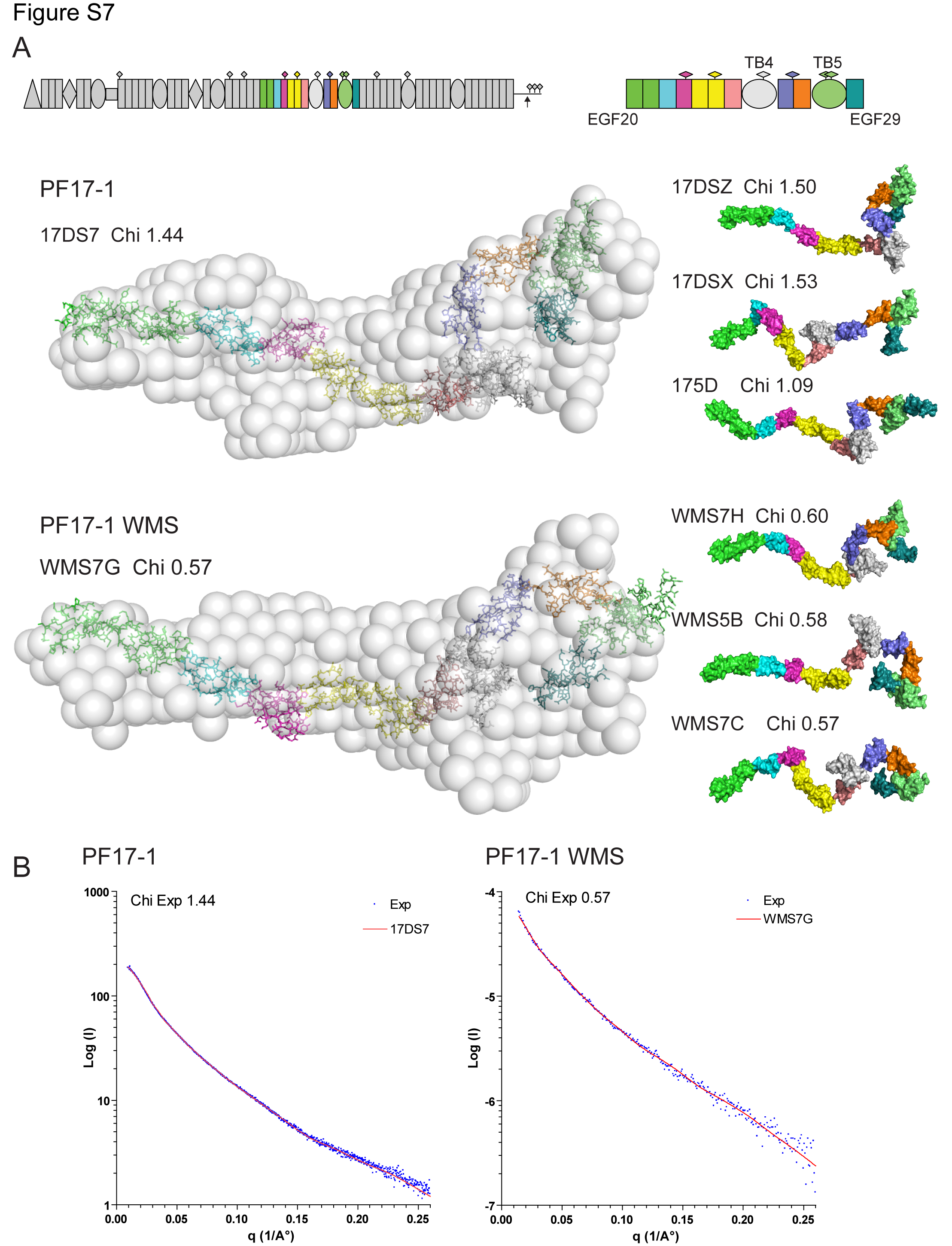

Supplement: Figure S7 — Rigid-body modeling of PF17-1 and PF17-1 WMS SAXS data. (A) Rigid-body models of PF17-1 and PF17-1 WMS were generated using SASREF using structural models of EGF20-21, EGF22, EGF23, EGF24-25, EGF26, TB4, EGF27, EGF28, TB5 and EGF29, as colored in schematic diagram of PF17. Shown are the best fitting models for PF17-1 and PF17-1 WMS, along with the most probable shape of the aligned models using DAMAVER and DAMFILT. Also shown are the 3 next best fitting models for each protein with their SASREF fitting error values. (B) Plots showing the experimental SAXS data plotted in blue as a function of q compared with the theoretical fit of the modeled structure with SASREF, shown in red. (TIF) [file pone.0048634.s007.tif]
