# Supplementary material for: Fibrillin-1 Mutations Causing Weill-Marchesani Syndrome and Acromicric and Geleophysic Dysplasias Disrupt Heparan Sulfate Interactions
Source: PLoS One. 2012 Nov 2;7(11):e48634. doi: 10.1371/journal.pone.0048634 (PMC3487758; doi:10.1371/journal.pone.0048634)
Supplement: Table S1 — List of oligonucleotide primers used in this study. List of primers used for domain swap constructs and site directed mutagenesis constructs. Restriction sites are shown italic. Domain overlap primers show amino acids encoded by the primer sequence. (DOCX) [file pone.0048634.s008.docx]

Table S1

|  | **FBN1 PF17-1 & FBN2 PF17-2 primer sets** |
| --- | --- |
| FBN1_Ex30-5' | GCGG *GTCGAC* ATC GAT GAG TGT GAA GAT AAT CC |
| FBN1_Ex43-3' | GGCC *AGATCT* CTA TTC ACA AAC CAA CAA CTT GTC ATT |
| FBN2_Ex30-5' | GCGC *GAGTCG* ACA TTG ATG AAT GTG AAA ACA ATC CTG |
| FBN2_Ex43-3' | AAGCTT *AGATCT* CTA TTC ACA AAC CAA CAG CAG GTC ATT G |
|  |  |
|  | **FBN1 and FBN2 Domain Swap Primer Sets** |
|  | FBN1-E28 G G N N C M D M R K S F FBN2-TB5 |
| F1E28-F2TB5_Fwd | AT GGG GGA AAT AAT TGC ATG GAC ATG AGA AAA AGC TTT TG |
| F1E28-F2TB5_Rev | CA AAA GCT TTT TCT CAT GTC CAT GCA ATT ATT TCC CCC AT |
|  |  |
|  | FBN2-TB5 H T G K A V D I D E C R FBN1-E29 |
| F2TB5-F1E29_Fwd | TT CAC ACA GGA AAA GCT GTT GAT ATT GAT GAG TGC CGG GA |
| F2TB5-F1E29_Rev | TC CCG GCA CTC ATC AAT ATC AAC AGC TTT TCC TGT GTG AA |
|  |  |
|  | FBN2-E28 G G N N C M D M R R S L FBN1-TB5 |
| F2E28-F1TB5_Fwd | AT GGA GGC CAC AAC TGC ATG GAT ATG AGA AGA AGT TTG TG |
| F2E28-F1TB5_Rev | CA CAA ACT TCT TCT CAT ATC CAT GCA GTT GTG GCC TCC AT |
|  |  |
|  | FBN1-TB5 Y T G L P V D I D E C K FBN2-E29 |
| F1TB5-F2E29_Fwd | TT TAT ACC GGT TTA CCC GTT GAC ATT GAT GAA TGT AAA GA |
| F1TB5-F2E29_Rev | TC TTT ACA TTC ATC AAT GTC AAC GGG TAA ACC GGT ATA AA |
|  |  |
|  | **FBN2 PF17-2 Mutant Primers** |
| FBN2_K1737R_Fwd | GGA CAT GAG AAG AAG CTT TTG CTA C |
| FBN2_K1737R_Rev | GTA GCA AAA GCT TCT TCT CAT GTC C |
| FBN2_K1737R_F1739L_Fwd | GGA CAT GAG AAG AAG CCT TTG CTA CCG AAG |
| FBN2_K1737R_F1739L_Rev | CTT CGG TAG CAA AGG CTT CTT CTC ATG TCC |
| PF17-2_K1770R_Fwd | CAT ATA ATG TGG GCA GAG CCT GGA ACA AAC CTT G |
| PF17-2_K1770R_Rev | CAA GGT TTG TTC CAG GCT CTG CCC ACA TTA TAT G |
| 172_+Q_I1794R_Fwd | TAA AAC CAT ATG TGG AAA TCA GCG TCC TGG ATT CAC CTT TGA C |
| 172_+Q_I1794R_Rev | GTC AAA GGT GAA TCC AGG ACG CTG ATT TCC ACA TAT GGT TTT A |
|  |  |
|  | **FBN1 PF17-1 WMS Mutant Primers** |
| PF17_WMS_Fwd | ATA ATT GCA TGG ATA TGA GAT ATG CTG ACA ACC AGA CC |
| PF17_WMS_Rev | GGT CTG GTT GTC AGC ATA TCT CAT ATC CAT GCA ATT AT |
|  |  |
|  | **FBN1 PF17-1 GD/AD Mutant Primers** |
| PF17_Y1696C_FWD | AGT TTG TGC TGC AGA AAC TAC |
| PF17_Y1696C_REV | GTA GTT TCT GCA GCA CAA ACT |
| PF17_Y1699C_FWD | TAC AGA AAC TGC TAT GCT GAC |
| PF17_Y1699C_REV | GTC AGC ATA GCA GTT TCT GTA |
| PF17_M1714_FWD | TTA TTC AAC AGG ACC AAG AAG |
| PF17_M1714_REV | CTT CTT GGT CCT GTT GAA TAA |
| PF17_G1726V_FWD | TAC AAC ATT GTC CGG GCG TGG |
| PF17_G1726V_REV | CCA CGC CCG GAC AAT GTT GTA |
| PF17_A1728T_FWD | ATT GGC CGG ACG TGG AAC AAG |
| PF17_A1728T_REV | CTT GTT CCA CGT CCG GCC AAT |
| PF17_S1750R_FWD | CTC TGT GGA CGT CAA AGG CCA |
| PF17_S1750R_REV | TGG CCT TTG ACG TCC ACA GAG |
